# What to take up from the patient’s talk? The clinician’s responses to the patient’s self-disclosure of their subjective experience in the psychiatric intake interview

**DOI:** 10.3389/fpsyt.2024.1352601

**Published:** 2024-06-21

**Authors:** Enikö É. Savander, Liisa Voutilainen, Jukka Hintikka, Anssi Peräkylä

**Affiliations:** ^1^ Department of Psychiatry, Päijät-Häme Central Hospital, Lahti, Finland; ^2^ School of Educational Sciences, University of Eastern Finland, Joensuu, Finland; ^3^ Faculty of Medicine and Health Technology, Tampere University, Tampere, Finland; ^4^ Faculty of Social Sciences, University of Helsinki, Helsinki, Finland

**Keywords:** psychiatric assessment interview, mental disorder, subjective experience, conversation analysis, third-position response, self-disclosure

## Abstract

During psychiatric diagnostic interviews, the clinician’s question usually targets specific symptom descriptions based on diagnostic categories for ICD-10/DSM-5 (2, 3). While some patients merely answer questions, others go beyond to describe their subjective experiences in a manner that highlights the intensity and urgency of those experiences. By adopting conversation analysis as a method, this study examines diagnostic interviews conducted in an outpatient clinic in South Finland and identifies sequences that divulge patients’ subjective experiences. From 10 audio-recorded diagnostic interviews, 40 segments were selected where patients replied to medically or factually oriented questions with their self-disclosures. The research focus was on the clinicians’ responses to these disclosures. We present five sequential trajectories that the clinicians offered third-position utterances in response to their patients’ self-disclosure of subjective experiences. These trajectories include the following: 1) the clinician transfers the topic to a new *agenda question* concerning a medical or factual theme; 2) the clinician presents a *follow-up question* that selects a topic from the patient’s self-disclosure of a subjective experience that may orient either towards the medical/factual side or the experiential side of the patient’s telling; 3) the clinician provides an *expert interpretation* of the patient’s self-disclosure of his or her subjective experience from the clinician’s expert perspective; 4) the clinician gives *advice* that orients mainly to a treatment recommendation or to another activity; and 5) the clinician presents a *formulation* that focusses on the core of their patient’s self-disclosure of his or her subjective experience from the patient’s perspective. In addition, we present what these responsive practices invoke from the patient in the next turn. We argue that an awareness of these strategies facilitates both the diagnosis and an appropriate therapeutic relationship during the psychiatric assessment interview. Finally, we discuss the clinical significance of our results regarding the patient’s agency and the clinician’s more conscious patient-centred orientation in the psychiatric assessment procedure.

## 1 Introduction

During the psychiatric intake interview, patients disclose their concerns regarding their symptoms, subjective problematic experiences, behaviours, feelings and relationships. Throughout their conversation with the clinician, they also give and take information and co-construct the diagnosis, rapport and treatment plan (1).

During the 1980s, the American Psychiatric Association (APA) as well as the World Health Organization (WHO) began to revise the contemporary categories of the DSM and ICD versions to advance the long-criticised reliability and validity of psychiatric diagnostic classification (2–5). Until this day, their successors are based on criteria and categories to determine a descriptive set of symptoms and behaviours. However, many authors have expressed concern that in addition to the descriptions of symptoms and behaviours that are needed for diagnosis, the patient’s subjective experiences in life circumstances and social relationships have been either neglected or received less attention. As a result, these consequences can adversely affect the validity of psychiatric diagnosis, individual treatment and empirical research (6–12).

A few decades ago, the principles of the patient-centred approach arose in medical and psychiatric institutions as opposed to traditional orientations that were doctor- or disorder-centred (13–15). Disclosure of the patient’s subjective experience in psychiatric interviews can be viewed as a pivotal moment when the doctor–patient interaction can move in the direction of being either disorder-centred or patient-centred. The patient-centred approach involves three core strategic elements, which are 1) communication, 2) partnership and 3) health promotion (16, 17).

Studies in psychiatric encounters report on the divergent priorities and orientations between patients and clinicians (18, 19). While the clinicians tend to orient to a diagnostic and medical agenda, the patients seek primarily to establish good relationships with their clinicians in an effort to receive a personal, empathetic understanding of their psychic conditions, diagnosis, prognosis and treatment (20–24). A good professional–patient relationship is a prerequisite for the patient’s active participation in their care. In outpatient psychiatric care, participation means being in and sharing information in reciprocal dialogue with professionals, being listened to and taking part in treatment activities and symptom management (25).

To describe the expression of personal experiences by patients, a Canadian psychologist, Sidney Jourard (26, p. 19), created the concept of self-disclosure and claimed that “*Self-disclosure is the act of making yourself manifest, showing yourself so others can perceive you*”. According to Jourard, self-disclosure advances healthy relationships and facilitates growth in the personal and social spheres of life. Another study, conducted by Elliot G. Mishler (27), investigated clinical medical encounters and likewise evaluated the participants’ expressions related to their lifeworld experiences and events. Mishler proposed a binary selection of ‘voices,’ the ‘voice of medicine,’ which involves a focus on medical and technical topics, and the ‘voice of the lifeworld,’ which references the personal meaning of events, experiences and life circumstances. Mishler claimed that when a patient adds some surplus content to their answer from their lifeworld, the sequential organisation of the interview is interrupted, causing gaps, hesitations or self-repairs as ‘troubles’ in the clinician’s next turn. In his time, Mishler claimed that the information exchange is performed through three-part sequences of the medical interview. He determined that the patient’s response is located between the doctor’s question and the assessment; in this manner, the doctor’s practices select and regulate the content of the conversation (27).

### 1.1 Psychiatric encounters in the field of interactional research

In psychiatry, the assessment of the patient’s psychiatric condition is based primarily on the conversation between the participant and the clinician’s observation of the patient’s behaviour. Unlike the somatic field of medicine, psychiatry has no particular technical tools or biomarkers that would advance the validity of diagnostic work. To understand the dynamics of psychiatric encounters, interactional studies examine psychiatric interviews from the perspective of both sides of the patient–clinician pair, and one valid method that is often adopted is conversation analysis (CA). CA is used to detect the structures and orders of naturally occurring interaction in different mundane or institutional social encounters (28–30). For example, McCabe and colleagues argued that divergent perspectives and orientations between interview participants might create ‘noticeable interactional tension’ and asymmetries, which affect a shared understanding and therapeutic relationship (31, p. 1148).

Studies on psychiatric interviews have analysed the clinician’s conversational practices and orientation to the patient’s answers. For example, one study reported on the ‘exploratively oriented style’ of clinicians, which entailed checking and clarifying their patient’s talk on their lived experiences as well as the manner in which the clinicians interpreted the meaning of experiences from the diagnostic perspective (32). Amongst others, one study on psychiatric interviews found that clinicians rarely attended explicitly to their patients’ emotional cues nor did they respond empathetically; nonetheless, they provided more space for concerns (including symptoms) when gathering diagnostic information (33).

Davidsen and Fosgerau (34) compared the responses of general practitioners and psychiatrists to the emotional disclosure of depressive patients. The authors detected different responsive practices in these institutional encounters. The general practitioners communicated emotional attunement without using formulations of the topic and considered their patients’ emotions within their life circumstances. By comparison, the psychiatrists responded to the patients’ emotional disclosure by interpreting their emotions, clarifying their symptoms and rational argumentation or changing the topic of conversation. The authors suggested that general practitioners approached patients’ depressive condition in terms of their patients’ life context, while psychiatrists were oriented more to the biomedical approach to depression as a disease.

The manner in which clinicians formulate their questions can have consequences for therapeutic relationships. Thompson and colleagues (35, 36) examined the declarative questions in psychiatric outpatient interviews. Declarative questions, which can also function as a formulation, are used in psychotherapy to target patients’ psychological and emotional gist of their experiences (for example: ‘*So you feel a bit anxious*’). The authors observed that practising more declarative questions in psychiatric interviews resulted in slightly better therapeutic alliances. Furthermore, researchers likewise reported that these declarative questions/formulations advance the topic transition empathetically and promote the progress of the interview as well as the therapeutic relationship in a patient-centred manner.

Recently, a CA study investigated a psychiatric assessment process with a patient with a personality disorder. The patient’s behaviour oscillates between engagement and disengagement in the interactions. While the patient’s dis/engagement might indicate emotional instability that is part of her disorder, the study also shows how dis/engagement is collaboratively produced in the interaction between the clinician and the patient (37).

### 1.2 Self-disclosures and third-position utterances

The present analysis qualitatively investigates one environment involving a clinician who responds in a third-position turn to a patient’s self-disclosure of subjective negative experiences. Antaki and colleagues (38) defined self-disclosure as a social action with three specific features. The first is that it is accomplished *voluntarily*. The second is that it is significant, very expressive and *emphasised*. Finally, the third feature is that self-disclosure describes intimate and *personal* experiences or additional information that departs from the momentary expectations of the participants. Kowalski (39) examined the consequences of self-disclosure of personally distressing information in clinical encounters. He showed that it is a fundamental interpersonal process related to well-being. Research in psychotherapy showed that patients reveal profoundly personal information; however, they might withhold several particular details; still, a strong therapeutic alliance facilitates disclosure, especially when the patients consider it important (40).

Concerning the case of a psychiatric interview, self-disclosure is the patient’s response to the clinician’s agenda questions, which are either medically or factually oriented. The patient’s response as a self-disclosure is made in the form of a surplus and voluntary account of their subjective negative experience. Yet occasionally, the clinician’s question serves a dual function, opening space for both the medical answer and the self-disclosure of a subjective experience. Using CA, we will examine how the clinician’s responsive actions—as third-position utterances—deal with the patient’s self-disclosure in this institutional context.

In general, *intersubjectivity* is a precondition for any social activity or even for personal well-being, and it is manifested in human interaction or communication (41). Indeed, John Heritage (42, p. 256) claimed that “*Linked actions, in short, are the basic building-blocks of intersubjectivity*”. The speakers thus impart knowledge to each other and display their pursuits or orientations turn-by-turn in the sequential organisation of their interaction. The relations between adjacent utterances therefore play a key role in the possibility of intersubjectivity.

Perhaps the most fundamental sequential organisation in conversation involves the *adjacency pair*, which refers to two consecutive utterances that relate strongly to each other. In adjacency pairs, the speakers display their understanding and intention of the previous speaker’s action (42, 43). As examples, consider a question and answer or a request and response in which the first pair part of this basic pair presupposes and anticipates the second pair part. The role of questions is also influential and powerful in each institutional interview (27, 44–47).

One aspect of the interview plays an equally important role in displaying an understanding of each other’s actions*—*the *third-position* action*—*such as the questioner’s reaction to the answer. The third-position action reflects the initial speaker’s perspective and understanding on the other’s reaction to his/her talk and thus affects, maintains or defends the intersubjectivity as well as ensures that the interaction moves forward (42, 48). In previous studies, the third-position action was observed to have a decisive and characterising institutional role in maintaining and moving the interaction forward in educational or medical and psychotherapeutic environments (49–51).

Using CA to investigate psychoanalytic encounters, Peräkylä (52) reported on how the psychoanalyst responds to the patient’s answers to the interpretations in the third-position utterances, imparting their professional knowledge and emphasising the gist of the patient’s description of concerns. In addition, Peräkylä presented a modifier role of the third-position utterances in a therapeutic larger interactional project. By investigating outcome interviews with patients in psychotherapy, Vehviläinen and colleagues (53) analysed the interviewers’ third-position responses to the patients’ telling of their experiences and events. These third-position responses involved repetitions, extensions, formulations and follow-up questions that were used to guide and shape the meaning of the patient’s account of their experiences.

### 1.3 Comparative study on the patient-centred approach

In their attempt to investigate and advance the patient-centred approach in the psychiatric assessment, Savander and colleagues (54, 55) compared the usual clinical interviews and assessment process with an alternative assessment process that is based on a psychological case formulation, and their comparison was conducted in a community mental health centre in South Finland. The current study is based on that same data set.

The alternative diagnostic process involves a psychological case formulation and is based on what is referred to as the dialogical sequence analysis (DSA). Developed by a Finnish clinical psychologist, Mikael Leiman (56), DSA is a microanalytical method to analyse utterances in freely flowing talk. The analytical unit of DSA is the speaker’s stance towards the referential object, which conveys a reciprocal relationship with the content (about what) and the recipient (to whom). In any social interaction, the words and non-verbal signs mediate a network of personal meanings and values that embody mental activity. By applying reflections or short formulations, a clinician can assist the self-observations of clients or patients and can identify regularities or patterns of maladaptive/habitual action that may affect and maintain symptoms and mental distress. The DSA method helps outline an individual psychological case formulation of a patient’s mental condition (57, 58).

Savander and colleagues (54) observed in their naturalistic comparative study that a clinician and patient arrive at a common perspective as a shared understanding of the treatment plan by focussing not only on diagnostical inquiries but also on both comprehending the patient’s individual maladaptive action patterns, which are referred to as obstacles to agency. In their results, during the visit for the treatment plan, the appraisals by the patients and clinicians were significantly more convergent in terms of the treatment goal, tasks and bond—the three parts of the working alliance (inventory) which are operationalised and quantifiable (59, 60)—in the DSA group than in the control group (AAU, assessment as usual). Furthermore, the period of the assessment procedure was shorter in the DSA process than in the usual psychiatric assessment process.

From this naturalistic comparative study, Savander and colleagues (61) moved on to adopt CA to compare five AAU interviews with five DSA-based interviews. The authors discovered that the DSA-based interview offered more opportunities for the patients to disclose their negative subjective experiences than the usual assessment interview. Thus, by guiding the patient to reveal more of their negative subjective experiences, the clinician attempted to inquire and respond to, or deal with, the patient’s topic and account. Researchers discussed that in this collaborative manner, the patient could take into account and conceptualise their emotional experiences as well as their description of their symptoms, advancing not only the diagnostic evaluation but their self-observation and active participation.

Savander and colleagues (62) conducted another analysis based on the data from their previous study (61). Using CA, the authors focussed on the patients’ interactional practices that involved the patients disclosing their subjective negative experiences as responses to the clinicians’ medically or factually oriented questions, that is, their agenda questions. The authors were able to determine four different trajectories in which the patients’ conversational control gradually increased. They observed that the patients formulated and performed their self-disclosures with many particular interactional practices that depicted the need and urgency of their account. For example, the patients used various means such as expressive words and idioms, extreme case formulations (ECF; 63), dramatisation of their stories, a loud voice, rhetorical questions, a complaining tone and an attempt to overtake the clinicians’ effort to continue their agenda. The researchers discussed that these repeated conversational actions revealed the interviewee’s occasionally divergent orientations, causing a mismatch and considerable interactional tension during the diagnostic interviews.

Building on these earlier studies, the current study examines the third-position responses to the patients’ self-disclosures in both AAU and DSA settings (case formulation).

### 1.4 Objectives

The aim of this study was to investigate 10 psychiatric interviews for the clinicians’ responsive actions in the third position which followed their patients’ self-disclosure of their subjective negative experiences. We examined what these responsive practices invoked from the patient in the next turn, and more generally, the ways in which the participants co-constructed the psychiatric interview.

## 2 Materials and methods

### 2.1 Participants and data

Our data were originally gathered for a randomised clinical trial conducted in a community mental health centre in South Finland (54, 55). The Ethics Committee of Tampere University Hospital approved the study in 2014. All cohort participants were required to submit their written informed consent. In the original comparative trial, all patients were randomised, and they were blinded to the style of assessment interview. Three psychiatrists and three psychologists were trained in the DSA approach, shaping the DSA intervention group. As a control group, the AAU group conducted a symptom-oriented descriptive diagnostic evaluation, usually done in public mental health care in Finland.

In this trial, 45 psychiatric intake interviews were audio-recorded with patients who had been referred to the mental health centre. These referrals were received primarily from either general or occupational physicians. For the purposes of the present study, 10 psychiatric intake interviews were analysed for a total of 563 min. The study used five randomly selected assessment as usual (AAU group) interviews based on the usual standard psychiatric interview (ICD/DSM) practice (280 min). These were matched with the five DSA-based assessment interviews (283 min) that were mentioned above in more detail (56, 58). The matching procedure of the two different interviews was based on seven clinical criteria that were performed in the earlier study. The matching process is based on the patient’s clinical characteristics, including 1) gender, 2) age, 3) educational level, 4) psychiatric treatment history, 5) substance abuse history, 6) the ability to self-reflect and 7) the ability to verbalise experiences, and these were mentioned in more detail in an earlier study (61). The researchers compared the two types of interviews quantitatively, focussing on the patient’s utterance of their subjective negative experience (61). From 10 recorded interviews, the authors selected the patient’s utterances of a subjective negative experience using non-medical terms (*N =* 119). The sequential organisation around those utterances was analysed and found to be medically oriented or experiential (non-medical) conversational environments that occurred before and after the utterances. From this comparative study, the researchers used CA to qualitatively analyse a further 40 sequences. These sequences consisted of the clinician’s questions that were medically or factually oriented and the patient’s self-disclosure of their subjective experience in response to the questions (62).

For the present study, we investigated the continuation of these 40 sequences from the previous study, targeting the clinician’s third-position responsive action and practices related to the patient’s self-disclosure of a subjective experience. The assessment interviews were unstructured, and the data were gathered from the history-taking or exploration part of the interviews. The clinicians possibly made some notes on paper during the interviews and they usually used a computer during the final part of the encounter to prescribe medicine, make formal statements or fill in forms. As in the original comparative study, our analysis excluded patients with a psychotic disorder or neuropsychiatric disorder, as well as anyone who needed urgent evaluation within 7 days. The participants in each interview consisted of the patient and two clinicians—a physician with a nurse or a psychologist. The data from five DSA-based interviews involved two psychiatrists, two psychologists with special DSA training and five adult patients. The data from five AAU interviews involved three nurses and one psychiatrist, two psychiatric residents and five adult patients. All patients with various symptoms and diagnoses participated.

### 2.2 Procedure

In this study, we further analysed the 40 sequential trajectories of the patient’s self-disclosure of a subjective negative experience that were detected and analysed by CA in the earlier study (62). As mentioned in the *Introduction* section of this article, CA is a qualitative research method used to investigate the speakers’ action sequences in mundane or institutional social interactions (28–30). The 10 interviews were transcribed using CA notation (64). The first author conducted a preliminary analysis of the data. She focussed on when and where the patient ended their self-disclosure, and the clinician provided their responsive action to them in the third position. Examining these sequences, the first and the fourth authors detected five different responsive actions performed by the clinicians. The five responsive actions formed five groups mentioned in the results. The first author analysed all the extracts in each group. Thereafter, the first and the fourth authors selected the most representative sequences from each group. The first author subsequently conducted an in-depth conversation analysis of these sequences. The next step for the first author was to negotiate and elaborate further on the analysis with the second and fourth authors. These sequences were presented in this article.

All personal information in our data has been anonymised in all extracts and examples. The indirectly identifiable data have been presented without age, gender and specific diagnosis. The original Finnish conversation data have been translated and are also presented in English.

## 3 Results

For this collection, from the 10 audio-recorded diagnostic interviews (five random AAU and five matched DSA), 40 extracts were analysed. These extracts contained medically or factually oriented questions that led to the patients’ self-disclosures. The focus of our research was on the clinicians’ responsive actions to them in the third position. We present five sequential trajectories as five groups, involving five different unequivocal third-position responsive activities (**Table 1**). We describe all groups and present representative extracts from each trajectory group. In the first group, the clinician listens throughout the patient’s self-disclosure and then shifts the topic to a new *agenda question* on a medical or factual theme. In the second group, the clinician presents a *follow-up question* that selects a topic from the patient’s self-disclosure of a subjective experience that may orient towards the medical/factual part or the experiential part of the telling. From this group, we present two examples in which the clinician moves the discussion towards medical/factual or experiential directions. In the third group, from a professional perspective, the clinician provides an *expert interpretation* of the patient’s self-disclosure of their experience. In the fourth group, the clinician produces advice that orients mainly to a recommendation for treatment. Finally, in the fifth group, the clinician presents a *formulation* that focusses on the core of the patient’s self-disclosure from the patient’s perspective. In all presented extracts/pictures, the focus line of the analysis is marked with arrows. In all extracts, the transcription symbols used were adapted from Jefferson (64; see also **Appendix**).

**Table 1:** Distribution of responsive actions to the patient’s self-disclosure of a subjective negative experience in the two different types of diagnostic interviews.

Responsive actions	AAU interview	DSA interview
1. Agenda question	8	–
2. Follow-up question	9	7
3. Expert interpretation	3	–
4. Advice	1	4
5. Formulation	1	7

AAU, assessment as usual; DSA, dialogical sequence analysis-based assessment.

The five types of third-position responses are related to the clinicians’ major lines of action. Almost in all cases, however, the clinicians’ responses also involved smaller, ‘subsidiary’ acts. Thus, in almost all cases, the clinicians provide space for the clients’ talk by contributing only minimal responses, continuers or acknowledgements, demonstrating that they listen to the patient’s account and understand it (65). In some cases, the clinicians offered short assessments (such as ‘*yeah, well, that’s understandable*’). After these types of moves, the clinicians could shift into the major third-position action. Furthermore, the clinicians could facilitate the patients’ telling by asking them follow-up questions before moving on to their major third-position action (see **Extract 3**, which features a follow-up question that precedes the formulation of the patient’s experience).

### 3.1 Agenda questions

The agenda question refers to the routine clinical questions during the history-taking and information-gathering phase of the usual medical or psychiatric interviews (45, 46, 53).

These agenda questions are usually interrogative turns that are medically oriented inviting symptoms or factual knowledge without the patient’s own lifeworld experiences, meanings and feelings. In these trajectories, the agenda question takes the place of a responsive turn to the patient’s self-disclosure and usually transfers the topic towards the medical or factual domain. We found that the clinician’s agenda questions usually began with a delay after the patient completed their account; however, these questions occasionally overlapped with the patient’s self-disclosure. One representative extract with a delayed response type from this group is the first extract.

**Extract 1** presents a middle-aged patient (PA) who has both a mood disorder and features of a personality disorder. This example is an extract from the history-taking part of the interview. Prior to this extract, PA discusses the burdens associated with dental care. Then, in lines 1*–*4, the clinician (DO) asks a complex agenda question concerning possible things that make PA happy or are sources of strength. This question has a dual function; on the one hand, it asks for factual knowledge of PA’s empowering factors; on the other hand, it allows PA to self-disclose subjective experiences. During lines 5*–*39, PA responds to DO’s question with an extended account of a self-disclosure. Firstly, in lines 5–6, PA initiates the turn with a “*noo/well*” preface, which may forecast the indirectness of the response and a need to negotiate the inquired topic, referring to the speaker’s personal perspective as a larger project (66, 67). Furthermore, PA uses the conditional mode here—”*well I could have*”*—*concerning things that are strengthening or sources of joy. Nonetheless, s/he subsequently shifts the topic to tiredness, answering contrarily, implying that PA is unable to accomplish these things. DO provides space by using a continuer (line 7), and PA begins talking about their appetite and weight loss. In the omitted data (lines 17–27), PA discusses in more detail about losing 15 kilos and sometimes eating fattening snacks. During PA’s extended response of self-disclosure, DO provides space for it and follows it with minimal responses or continuers.

**EXTRACT 1**

**Figure:**
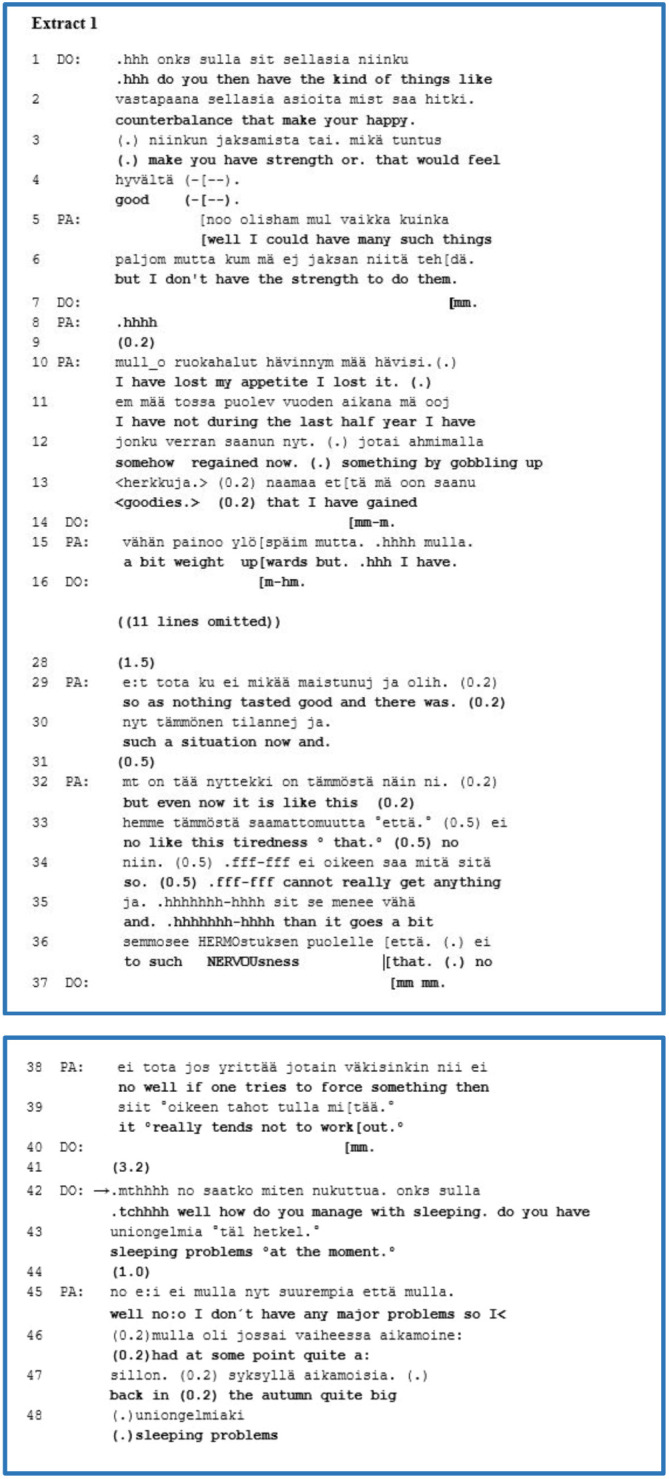

During the final part of the self-disclosure of a subjective experience (lines 32–39), PA offers an account of the meaning of tiredness and nervousness at present. PA emphasises their import through ECF by taking a long in-breath, sighing and alternating the volume of the talk, and these legitimise their concerns and complaints as being urgent and significant (62, 63).

Our research focus is on the clinician’s response to the patient’s self-disclosure. In line 40, DO provides a minimal response and, after a long gap (3.2 s), presents a new agenda question concerning sleep problems. Presenting a long gap in the timing of a response may refer to the speaker’s dispreferred or rejected action towards the previous speaker’s account (68). A preface*—*”*.mthhh no/.tchhhh well*”*—*creates a bridge and may predict and guide a shift to another topic, implying DO’s symptomatically oriented new question in lines 42–43 (66, 67). After a new gap (1.0 s), PA answers with the new topic of sleeping problems. However, PA also begins the turn with a preface of “*no/well*”, forecasting a further account from their own perspective and a need to negotiate.

In this extract, we found that the clinician took a break with a long gap and used a well-preface at the beginning of the agenda question in line 42. While the patient’s self-disclosure indicated that it is significant and urgent for the patient, the clinician neither treated it nor affiliated with it; in other words, the clinician rejected it and adopted no stance towards the experience.

While our focus is on the third-position action, we can nonetheless acknowledge that the patient also used well-prefaces at the beginning of their answers twice, in lines 5 and 45. It appears that both participants used conversational practices with which they competed to express their own perspectives, implying interactional tension between them. In another case in this group, the clinician’s responsive turn overlapped with the patient’s account and consequently shifted the topic to factual or medical issues. Overlapping in the turn initiation may imply that the answer is designed and created from the speaker’s own perspective (69, 70). In other words, by presenting the agenda question, the clinician used conversational practices that facilitated performing it. During the patients’ turns, the clinicians usually listened and responded to their patients’ self-disclosures of subjective experiences by using minimal responses or continuers.

In this group, we analysed eight extracts and focussed on the moment when the patient ended their account and the clinician presented their agenda question as a third-position response. We observed that the clinicians engaged in various interactional practices. For example, they could take a break—a gap—and use a well-preface before posing their agenda question or overlap with the patients’ account. These practices enabled the clinicians to withdraw themselves from the patients’ perspective of an experience or compete with it to maintain their agenda setting of the interview. These interactional practices imply considerable tension between the participants momentarily, conveying divergencies in their larger interactional projects and orientations.

### 3.2 Follow-up questions

In our collection, we discovered 16 extracts in which the clinician asks something further regarding the patient’s self-disclosure of a subjective experience. These follow-up questions select the topic from the patient’s account or check the interviewer’s presuppositions or perceptions (45, 53). These follow-up questions are also occasionally wedged between the patient’s account, interrupting the flow of the telling momentarily or located at the end of the self-disclosure. We determined that these interactional responsive turns steer the interview in the direction of medical/factual or experiential orientation.

In **Table 2**, we present the distribution of the different orientations of the follow-up questions in the AAU and DSA interviews. Both interviews contained both types of orientations of follow-up questions. Nevertheless, follow-up questions with the medical/factual orientation occurred more in the AAU than in the DSA interviews, in which follow-up questions with both orientations occurred almost equally. Next, we present two representative extracts. During the first, the clinician’s follow-up question directs the interview towards medical or factual orientation (**Extract 2**). In the second, the follow-up question addresses and topicalises the patient’s subjective experience (**Extract 3**).

**Table 2:** Distribution of different orientations of follow-up questions to the patient’s self-disclosure of subjective negative experience in the two different types of diagnostic interviews.

Follow-up questions	AAU interview	DSA interview
1. Medical/factual	8	3
2. Experiential	1	4

AAU, assessment as usual; DSA, dialogical sequence analysis-based assessment.

#### 3.2.1 Follow-up question with medical/factual orientation

The patient (PA) has a recurrent mood disorder, and this extract is from the history-taking phase of the psychiatric interview. At the beginning of **Extract 2**, the clinician (DO) refers to PA’s third depression phase and inquires as to when that phase occurred (lines 1*–*4). PA confirms the timing of DO’s polar question in line 5 and continues the topic regarding the challenging work–life experience. Lines 9*–*20 are omitted; within those omitted lines, PA spoke about his/her present problem of experiencing strong extreme emotions and frustration as well as his/her previous challenging job during her recent depression. In line 22, PA continues to define the timing of his/her current depression phase, but s/he jumps forward to the present to exemplify his/her “*really strong*” feelings recently last Tuesday. PA describes the vivid experience of anxiety experienced at work, as “*£ I just wa(a)nt to ge(e)t o(o)ut of here*” (lines 26*–*27) and “*I want to die*” (line 29), which imply the relevance and urgent meaning of the self-disclosure. From line 34, PA shifts the topic back to the previous depression phase, which was “*so tough*” at work, and at that time, PA consulted a psychiatrist who prescribed antidepressants.

**EXTRACT 2**

**Figure:**
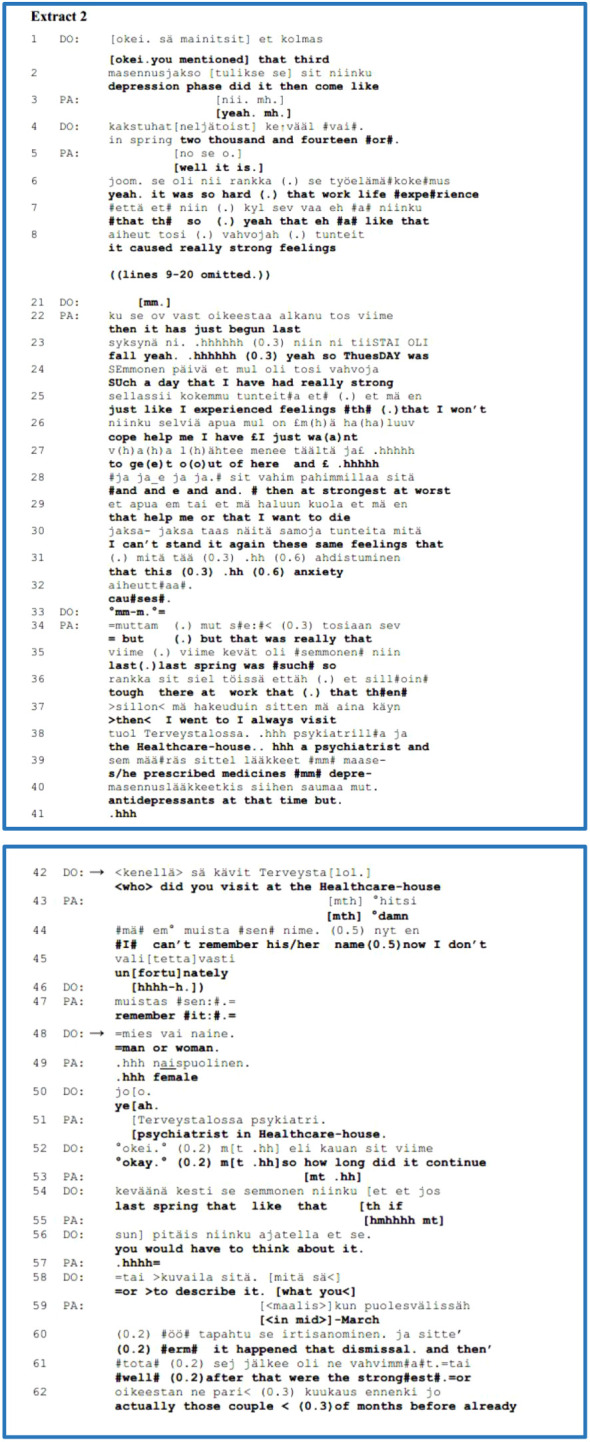

In lines 40–41, PA completes the account with “*at that time but.*” and then takes an in-breath. The conjunctive particle of *“mutt./but.*” as well as the in-breath may imply that PA intends to continue the turn; however, the final falling intonation suggests that s/he has completed his/her account. Immediately, in line 42, DO asks a follow-up question regarding the psychiatrist’s name, but PA does not remember it *“unfortunately*”. In line 48, DO poses another follow-up question concerning the psychiatrist’s gender, and PA answers it as a “*female*” psychiatrist in the healthcare house. In the next line, 52, DO acknowledges PA’s answer with a quiet “*okay*” and continues with a third complex follow-up question on how long the previous depressive phase lasted and invites a description of it, which implies factual knowledge as well the PA’s experiences.

These follow-up questions select and steer the topic from the patient’s account of their experience towards factual knowledge, which was important to the clinician for some unknown reason. Here, the patient’s vivid and emphasised description of extreme feelings and anxiety, including the thought of death, has neither been approached nor responded to by the clinician.

#### 3.2.2 Follow-up question with experiential orientation

**Extract 3** features a young adult patient (PA) with a mood disorder who makes a self-disclosure during the history-taking part of the intake interview. This extract involves clinicians, a psychiatrist (DO) and a psychologist (PS), who come together to meet PA. Prior to this extract, by referring to PA’s former telling, PS inquired about the patient’s previous intention to seek help from a psychiatrist or psychologist and when did it occur. PA replied that it was a half year ago. PS then asked what PA thought of personal problems at that time and what such a visit should provide. PS’s inquiry is complex. On the one hand, it approached factual knowledge, and on the other, it left spaces to present PA’s knowledge of problems or experiences. In the answer, PA no longer remembered why s/he had wanted to see a psychiatrist. S/he also recalled that s/he looked at the prices of the private sector and then decided not to go.

PA continues the account by initiating a new topic on his/her subjective experience as a self-disclosure. PA displays a tendency to downplay personal problems, comparing it to a habit of PA’s mother (lines 1, 3–4). PA sniffles and sobs; s/he also uses a metaphor expressed with faster talk than the surrounding talk, as “*isku vasten kasvoja*” which translated as “*like a slap on the face*”, emphasising the urgent meaning of the disclosure (lines 5, 7). PA’s account conveys that s/he realised that this personal habit of downplaying is what made the boss insist on PA seeking help from occupational healthcare (lines 7, 9, 11). During PA’s account, both clinicians follow it up with continuers.

**EXTRACT 3**

**Figure:**
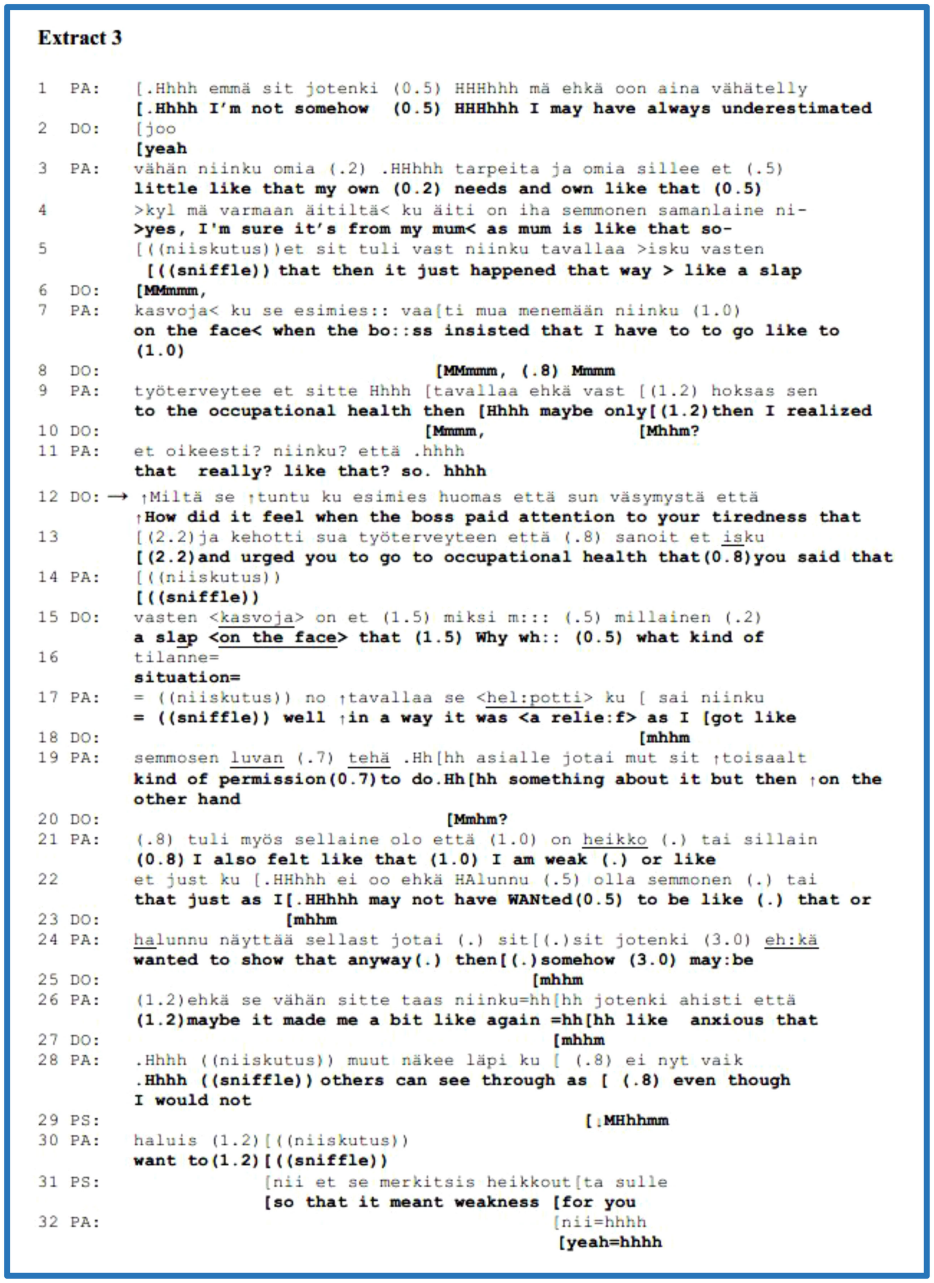

PA’s disclosure trails off at the end of line 11 with the final conjunction, ‘*että./so.*’. This conjunctive particle may leave open some implicit meaning of the previous account while ending the turn (71).

If we look more closely at our research focus in lines 12*–*16, DO presents a follow-up question approaching PA’s experience and feelings about the situation when the boss ordered PA to request help. S/he recycles the metaphor from PA’s previous disclosure as “*like a slap on the face*” and considers and maintains PA’s topic and perspective, inviting further elaboration. In lines 17*–*30, PA continues the disclosure and elaborates on their experience. PA begins to answer directly by sniffling and a ‘*no/well*’ preface telling the account from a personal perspective and a need to negotiate. PA states that the boss’s order made it easier to be weak. PA subsequently advances this topic and completes the account about anxiety or fear that “*others can see through*” that. In line 31, the other clinician (PS) poses a declarative question or formulation (35), also matching as a follow-up question to the patient’s experiential realm. Dealing with the self-disclosure of a subjective experience ends with the patient’s confirmation of “*yeah=hhhh*”, joining the participants’ mutually achieved perspective. In addition, we can take into account that the clinician’s follow-up question with an experiential orientation prepared the ground for the clinician’s next formulation of the patient’s experience (line 31).

To summarise, for this group, we analysed the function of follow-up questions as third-position responses to the patient’s self-disclosure of a subjective experience. We found that the follow-up question can steer and control the progress and direction of the interview. In our representative **Extract 2**, the follow-up question, as a responsive action, directed the conversation towards a factual matter that the clinician was interested in. In **Extract 3**, the follow-up question, as a responsive action, dealt with the patient’s perspective and attuned their self-disclosure of a subjective experience, advancing their further elaboration. We occasionally came across follow-up questions (not presented here) that immediately took up some information from the patient’s account, but after the patient’s brief answer, the previous topic was able to progress further.

In **Table 2**, we presented the distribution of two different orientations of follow-up questions to the patient’s self-disclosure of that patient’s subjective negative experience in the two different diagnostic interviews. In these relatively small data, we discovered that the clinicians in AAU interviews asked additional follow-up questions that directed the information exchange towards factual or medical matters. By comparison, the clinicians in DSA interviews almost equally approached their patients’ self-disclosures with follow-up questions regarding their perspectives on subjective experiences and medical/factual knowledge.

### 3.3 Expert interpretation

The psychiatric literature and psychotherapeutic interactional research describe and address interviewing techniques and this includes interpretations (52, 72–75). Interpretation is an act that explains what is not immediately apparent and conveys an interpreter’s or expert’s conception. In our collection, an interpretation represents a clinician’s responsive statement from their expert perspective and knowledge concerning the patient’s account.

Conversational analysts in psychotherapy research argued that the therapist’s interpretation challenges and invites the client to alter their personal perspective of the experience or event. The clients respond to the interpretation with matched or mismatched responses in some manner. For example, the client’s simple acceptance of the interpretation by an acknowledgement might be dubious or unreliable; nonetheless, an ‘extended agreement’ with longer elaboration refers to active contribution (76–78).

We will demonstrate that during psychiatric assessment interviews, the clinician’s interpretation may have different consequences than in psychotherapeutic encounters. In our analysis, these interpretations are third-position responses that convey symptomatic or diagnostic and generalising explanations from the clinician’s perspective concerning the patient’s account of a problematic experience. We detected three extracts with the clinician’s interpretation of a patient’s account of self-disclosure.

The next example to consider is from **Extract 4**, in which the clinician (DO) offers a symptomatic or diagnostic explanation for the patient’s (PA) self-disclosure of a subjective experience; moreover, a nurse (NU) also gives a presentation during the interview. This is a young adult patient with anxiety and unspecific stomach pain. This part of the interview is from the explorative phase when DO presented, amongst others, two familiar agenda yes–no questions concerning paranoic or psychotic symptoms. In response to the first question (line 1), PA gave a direct negative answer, and DO immediately moved on to the next agenda question regarding the experience of thought reading (lines 4–6). By overlapping DO’s second question, PA disclosed a negative experience and feelings “*during a youth meeting*” with friends in a particular manner in lines 7–19. For example, s/he uses the ECF of “*even a little*” (line 13) and has a creaky voice (79) in lines 15 and 19 that can convey turn transitions; these also appear to be associated with sadness in this context. PA’s account is not an invited direct answer, but an extended self-disclosure of a problematic experience with affective features that s/he is “*looked at in a bad #<way.>#*” by friends. DO actively followed the extended answer with acknowledgements in lines 12 and 17, while PA completed the account in line 19.

**EXTRACT 4**

**Figure:**
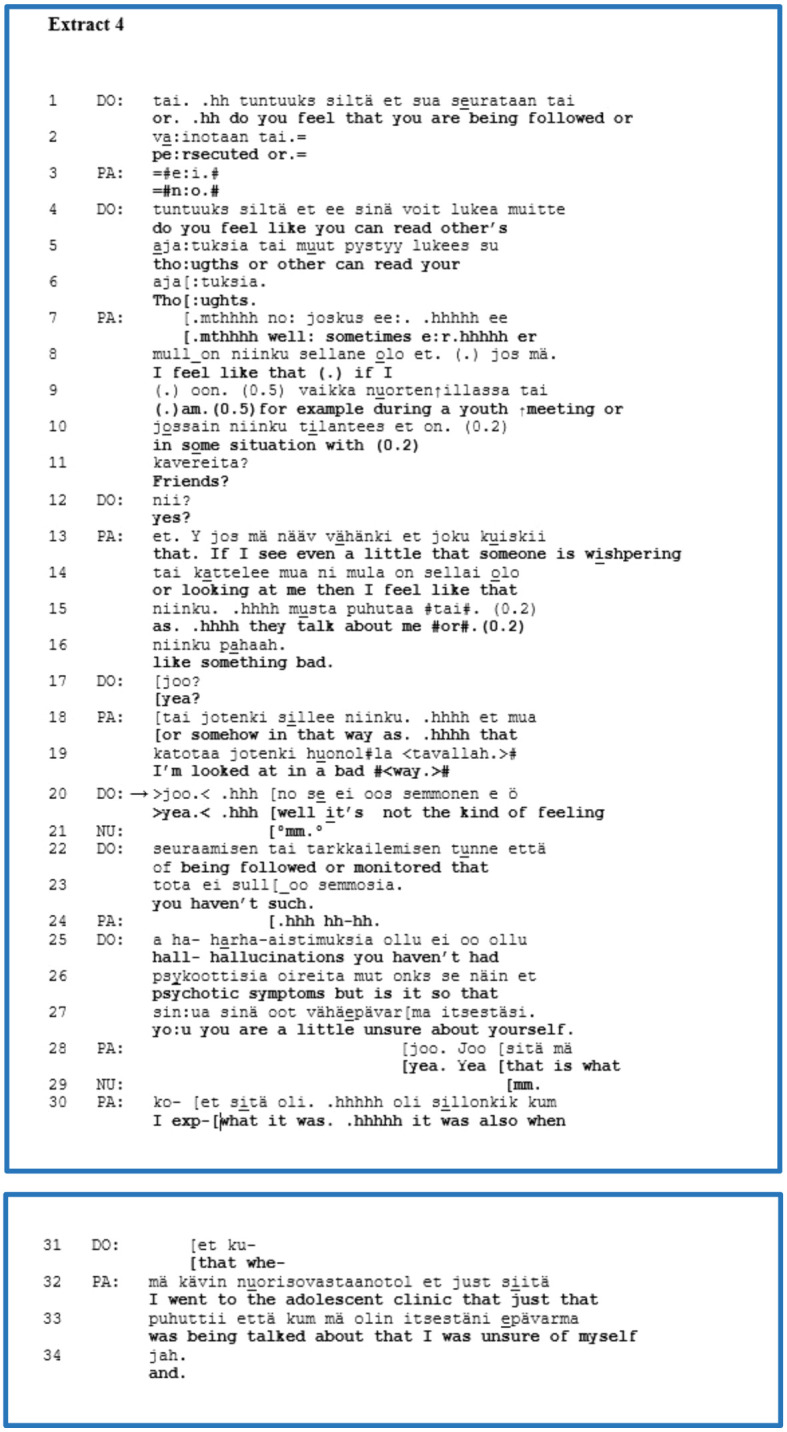

Our analytical focus is on DO’s third-position interpretation in lines 20–27. Firstly, DO received PA’s account with an acknowledgement directly and after an in-breath; s/he began the statement with a ‘*no/well*’ preface that forecasted the subsequent account from a personal perspective. At this point, DO assesses PA’s experience as neither a hallucination nor a psychotic symptom. Naturally and apparently, PA might be unaware of whether or not the experience is a symptom, that is, whether it is diagnostically relevant. Immediately following the symptomatic interpretation, by using an adversative conjunction, “*but*”, DO shifts the topic by posing a yes–no question in response to PA’s characterisation or a sort of label in lines 26–27—”*a little unsure about yourself*”—offering another possible explanation or interpretation for PA’s experiences. Moving on to line 28, by overlapping DO’s turn completion of the question, PA preferred and answered in the positive to the question immediately, using repeated acknowledgements, and elaborated on the topic related to PA’s previous experience in the adolescent clinic upon hearing from the experts/clinicians of his/her being unsure. The patient elaborated on his/her previous experience in youth mental healthcare by providing an extended agreement.

To summarise, the clinician responded to the patient’s self-disclosure of a subjective problematic experience from the symptomatic/diagnostic perspective, offering an expert interpretation. In this example, the clinician first offered a diagnostic interpretation followed by an explanation of the patient’s “*unsure*” character; thereby, s/he was neither affiliated with the patient’s perspective of experience nor approached it. Thus, the clinician selected and differentiated the patient’s problematic experience, being or not diagnostically relevant symptoms and offered an alternative interpretation of that person’s experiences. In this case, the patient provided an extended agreement during his/her elaboration on a previous personal experience in youth mental healthcare. This group also had two other cases (not presented here) for which the clinicians offered their expert interpretations of their patient’s experience and immediately, in the same turn, transferred the topic to another agenda question without waiting for their patient to respond. The clinician in this small group used third-position interpretations that predominantly served the clinician’s diagnostic evaluative work and performed different functions than in psychotherapy. As shown in **Table 1**, all three extracts that had interpretation were found in the AAU interviews.

### 3.4 Advice

As a psychiatric interviewing technique, advice constitutes an expert device, being acceptable and desirable in this institutional encounter. However, the psychiatric educational literature warns that premature advice can be inappropriate and intrusive if the doctor does not provide sufficient space and time to listen to the patient’s problem (72).

Furthermore, interactional studies in different institutional contexts have demonstrated that advice-giving as a problem-solving action, on the one hand, delivers an expert’s viewpoint and plays a central role in an institutional encounter; on the other hand, the perspective and concern of the client or patient may go unnoticed, weakening their agency. Researchers have pointed out the dilemma of advice-giving and have suggested that it should be produced collaboratively with a client or patient, balanced with information-giving, support and empathetic understanding. Indeed, when providing advice and offering a different way of thinking or behaving that the other is unaware of, this creates an expectation that it will be accepted or rejected (80–84).

We discovered five instances of clinician advice-giving in our data that were their third-position responses to the patient’s self-disclosure of a subjective experience. Our representative example from this group is **Extract 5**, which features an adult patient (PA) with a mood disorder and difficulty in managing aggression. Two clinicians are present in this extract, a physician (DO) and a psychologist (PS). Before this extract, PA revealed that s/he works with his/her father in their family business and complained about the father’s tantrums. In lines 1 and 4, DO poses a yes–no question concerning any violence on the part of the patient’s father, which is a psychiatric agenda question pertaining to risk behaviour. PA overlaps with that question in line 2 and apparently begins a confirming answer but leaves it unfinished. In line 5, by overlapping with the end of DO’s question and by using a well-preface, PA denies that his/her father ever exhibited violent behaviour. In lines 6–13, PA elaborates a self-disclosure regarding mental abuse and characterises the father with the metaphor “*such a tyrant*”. During PA’s account of a problematic experience, DO follows PA’s telling with acknowledgements and minimal responses actively (lines 8, 12), closing the sequence with “*mm. (.).mt[yea:*,” in line 14.

**EXTRACT 5**

**Figure:**
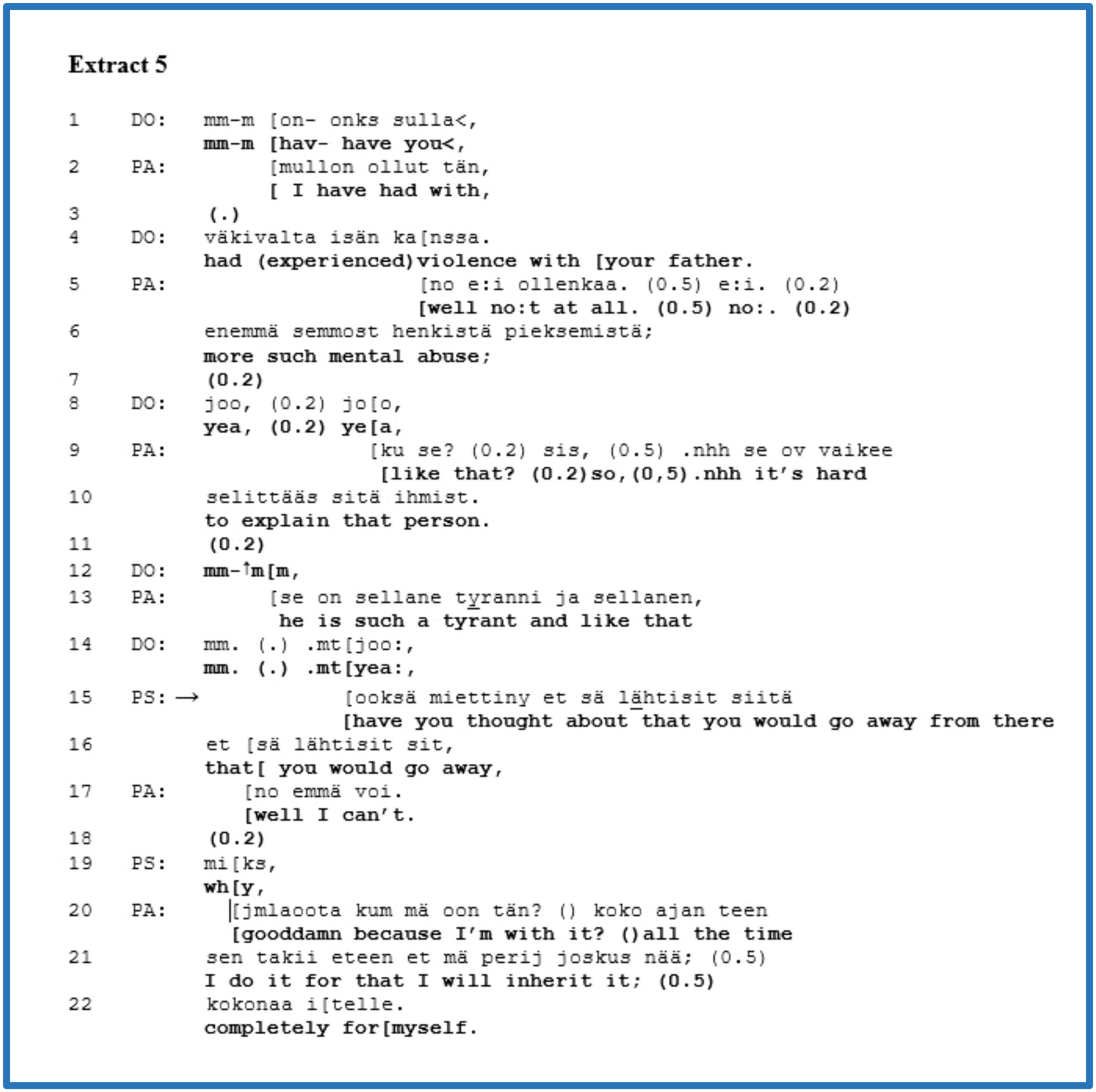

At this point, let us focus on the turn-taking of PS, which overlaps with DO’s acknowledgement. PS’s advice is formulated as a yes–no question in the conditional (lines 15–16). PS suggests leaving the family business and emphasises the account by repeating the phrase twice. In line 17, by overlapping the previous turn of PS, PA presents a quick, negative answer beginning with a well-preface, which reflects the problematic nature of the proposed advice. PS continues with the follow-up question, “*why*”, inviting an explanation regarding PA’s decision to remain in the family business. In 20, directly, PA’s turn begins with a swear word as an expletive, expressing his/her stance with a negative emotion or verbal aggression towards the escalating situation and discloses that s/he still intends to inherit the firm. The use of a swear word conveys an intent to invoke affiliation from the hearers (85).

This extract presents the clinician’s advice in a third-position turn that follows the patient’s self-disclosure of a subjective experience. The clinician offered a solution from their own perspective to the escalating situation between the patient and his/her father in the family business. We can ask whether or not the clinician’s advice was premature. According to the patient’s responses, firstly, the patient’s immediate negative answer in line 17 and, secondly, the explanation that contains a swear word beginning in line 20 conveys that the clinician’s advice was possibly premature. The clinician did not treat the patient’s perspective because s/he did not know enough about it. Regardless of the patient’s opposing positioning to the clinician’s advice, the patient displayed active participation and agency at this moment in the interview.

We found five extracts in this group. For other extracts from this group, when clinicians did not deal with the patients’ self-disclosure of a subjective experience, while giving advice, clinicians recommended a solution that worked for those patients in the past. There was only one extract where the clinician first offered a short formulation of the patient’s experience of tiredness and subsequently directly asked about matters such as the possibility of childcare. While in our representative extract, the patient rejected the clinician’s advice, in the other four extracts, they accepted and elaborated on the advice given in some manner, which means that advice-giving fulfilled its institutional function during those moments. There were four extracts in the DSA-based interviews and one in the AAU interview.

### 3.5 Formulation

One recommendation in the psychiatric educational literature is to use reflection as an interviewing technique. This means that the clinician empathetically paraphrases what the patient is trying to say. This has two types of function; on the one hand, the clinician indicates that s/he listens to the patient, and on the other, the clinician understands their account and concerns (72).

Many decades ago, Carl Rogers, the father of client-centred psychotherapy, claimed that the patient’s response to the therapist’s interpretation or clarification typically resists their self-exploration; nonetheless, “*Reflection of feeling by the counselor is followed, more often than would be expected by chance, by continued self-exploration or insight.*” (86, p. 321). To Rogers, understanding another individual’s perspective meant entering entirely and empathically into their frame of reference. Interactional research defines formulation as a speaker’s response to the gist of the other speaker’s prior experiential account in everyday or institutional conversations (87–89). The formulation is reminiscent of the reflection, the client-centred therapeutic or psychiatric interview technique. By using a formulation, the clinician offers a candidate understanding and focusses on or refers to the gist of the account from the patient’s perspective. It might subtly select, correct and transform the patient’s telling for a therapeutic or institutional purpose, all the while maintaining the speaker’s own perspective. Researchers have argued that the therapist’s interpretation invokes the challenging task of the patient to decide their position on an expert viewpoint. Instead, the formulation might advance the patient’s sense and meaning of their own previous experiential account, inviting confirmation or disconfirmation and affecting their position towards themselves or their self-observation (78, 88–92).

This group had eight extracts with formulations. Turning our focus to a longer trajectory with the clinicians’ formulations, the first formulation prepares the way for the next more extended formulations of the patient’s psychological activity. **Extract 6** is from a DSA-based assessment interview with a young adult patient who has a mood disorder. A psychologist (PS) and a psychiatrist (DO) with a patient (PA) participated in this interview. This is from the history-taking part of the interview in which PS asks about the patient’s “*social circle*” as a complex agenda question (lines 1–5) that allows a more extended account rather than a mere list of close persons. During the interview and the extract, PA cries a little or sobs, which is audible in PA’s sniffles. At the beginning of the interview, PA states that lately, s/he cries easily. As a non-lexical sound, PA’s sniffling may indicate a troubled state of mind or emotional distress (93, 94). From lines 6 to 26, PA provides an extended answer about mates and close friends and discloses experiences with a particularly close friend. PA’s presentation of that close person embodied the features of self-disclosure; the volume of PA’s speech is louder than the surrounding talk “*TALKED MORE*” (line 20) and uses ECFs such as “*really big help*” and “*always tries to help*”, emphasising the close person’s personal description (marked in blue in lines 22, 24 and 26).

**EXTRACT 6**

**Figure:**
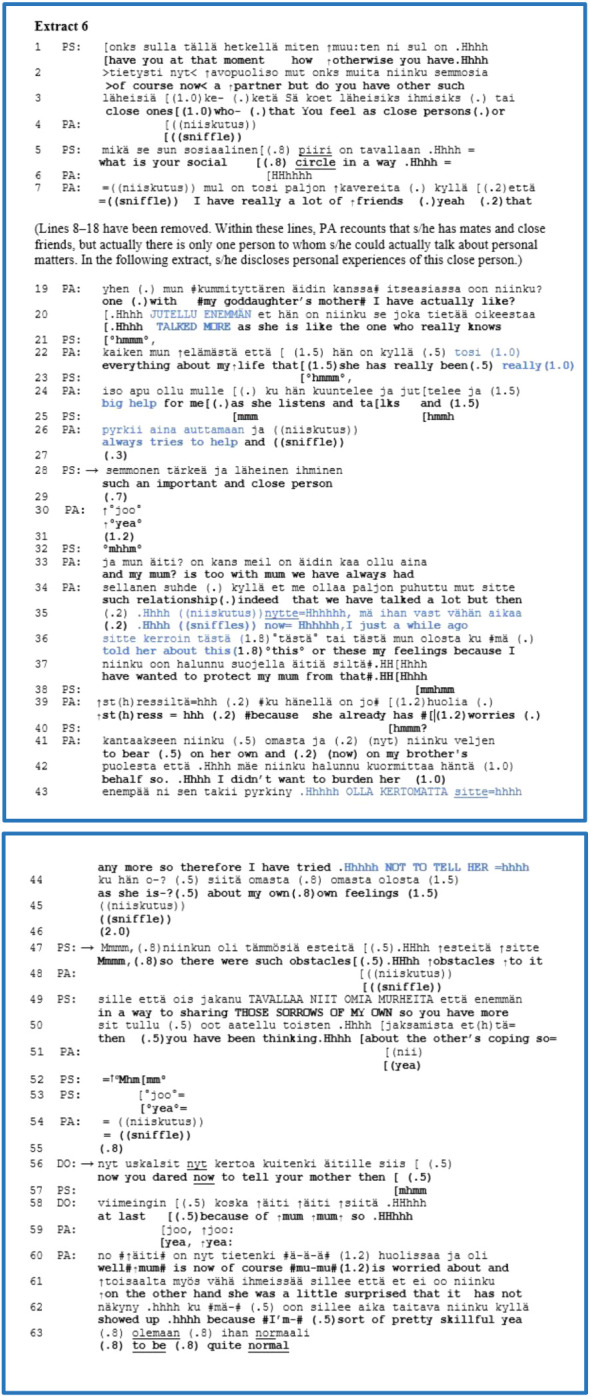

Our focus is first on line 28, which is when PS responds relatively fluently with a formulation. PS considers PA’s expressions—such as the use of ECFs and PA’s loader speech volume (lines 20–26, marked in blue)—as well as the meaning of PA’s disclosure, referring to the close person to confide in. PS formulates the important relationship from PA’s perspective. After a small gap (line 29), PA confirms that formulation with an acknowledgement that has a lower speech volume than the surrounding talk (line 30). PS provides space with a relatively long pause (1.2 s) and a silent minimal response in lines 31–32.

From lines 33 to 45, PA moves on to elaborate on the relationship with his/her mother. At the end of this self-disclosure, PA reveals that s/he did not want to burden her with troubles and feelings, emphasising the account with a louder volume of talk as “*NOT TO TELL HER*” (line 43, marked in blue).

Then, from line 47 to line 50, after a 2.0-s gap, PS again offers a slightly longer formulation, describing PA’s usual manner of acting and relating to others. PS names PA’s activity as a psychological obstacle, maintaining the perspective of the previous account. However, PS’s formulation transforms and generalises the meaning of the topic of disclosure from relating to the mother to relating to others. This transformation might have been generated from previous interview utterances on the same type of topic (not shown here). By overlapping with the end of the formulation, PA provides a short agreement, “*yea*”, in line 51.

In lines 52–53, PS’s continuer and acknowledgement with a small gap also pave the way for DO’s formulation (lines 56–58). DO formulates and takes into account PA’s former utterance from the same disclosure in lines 35–36 (marked in blue) that s/he only recently talked to the mother about feelings and concerns. PA’s utterance indicates that s/he has told personal feelings to the mother “*just a while ago*”. The adverbs of the time indicate that s/he might be slightly embarrassed or engaging in self-blame concerning his/her mother because s/he should have shared the affairs earlier. During this utterance, PA”s behaviour is divergent because at this point, s/he shared concerns and did not withdraw regardless of the embarrassed stance towards themselves. DO’s formulation selects the divergent behaviour from the same disclosure by the patient and from which PS generated the previous formulation. Reciprocally, by attempting to balance PA’s negative attitude towards themselves, DO’s formulation (lines 56–58) focusses on the behaviour that PA “*dared*” to tell his/her mother “*finally*” regarding personal feelings which maintain a divergent and more positive stance to PA than PA’s stance to themselves. PA’s confirmation with double acknowledgements overlaps with DO’s account; thus, DO’s formulation remains unfinished. Immediately, PA moves on and elaborates on the mother’s surprised reaction to PA’s telling of concerns because s/he has a “*pretty skillful*” habit of presenting themselves as “*quite normal*”, regardless of troubles and sorrows.

Thus, in this trajectory, we present three formulations but they are of two different types. The first short formulation provides preparatory work 1) by displaying understanding for the subsequent more extended formulations and the others and 2) by offering explanations of the patient’s usual behavioural pattern with others (50). The second formulation focussed on the patient’s habitual activity of withdrawing from others and protecting them. Nonetheless, the third formulation noticed the patient’s opposite activity, enabling them to share. Thus, the third formulation attempts to transform the patient’s relation positively to themselves as well as to the patient’s mother. We demonstrated that the patient agreed with the clinicians’ formulations and further elaborated on them. The three formulations cooperate step-by-step with the patient’s self-disclosure and might momentarily advance the patient’s self-observation collaboratively as well as the working relationship.

This representative extract is from a DSA-based assessment interview in which the clinicians are trained in DSA theory and concepts. By orienting to the analytical unit of DSA—the speaker’s stance or relation towards the referential object—the clinician may use formulations to define the gist of the patient’s knowledge and perspective on the experience. In this case, the second and third formulations mirrored and embodied the patient’s two divergent behaviours that might manifest a psychological conflictual dilemma between sharing concerns and a withdrawal from telling them.

To summarise, our representative extract of this group revealed that formulation invited the patient to agree and further elaborate. Eight extracts were found, of which seven were from the DSA interviews and one from the AAU interviews. In the case of the AAU interview, the nurse offered a formulation which displayed understanding about the patient’s self-disclosure: “*mm a little sadder feeling.hhh you often feel sad like that*”.

The patient agreed with the clinician’s formulation and elaborated on the topic in five extracts, which resembled the representative extract. However, three of the eight extracts had dubious responses in which they did not resist the formulation; instead, they recognised the topic and elaborated on it from their own divergent knowledge and perspective. For example, one extract featured the patient offering the following repair response to a formulation: “*so could it be that the last energies have been squeezed into the things that have to be done*”. The adverb “*nii/so*” offered a recognition or assessment of what the previous speaker said that is not an explicit agreement (95). Furthermore, in two of the eight extracts, after the patient’s agreement that was achieved through an explicit acknowledgement or minimal response, the clinician took their turn by posing a follow-up question related to the same topic.

## 4 Discussion

This study analysed interview sequences in which, after the clinicians’ medically or factually oriented questions, the patients self-disclosed their negative subjective experiences. The precise focus of our analysis was on the clinicians’ third-position responses to the patients’ self-disclosures. We detected five different response types based on how the third-position responses dealt with and managed the patients’ self-disclosures.

We presented representative extracts from the five response types. These response types form a continuum based on whether the clinician either maintains distance from the content of the patient’s prior self-disclosure or moves closer to it. At one end of the continuum stands the agenda questions that re-establish the clinician’s expert rights and knowledge and that direct focus away from the experiences disclosed by the patient. At the other end lie the formulations that legitimate the patient’s self-disclosure and consider its content from the patient’s perspective. Between the extremes are other response types—follow-up questions, expert interpretations, advice—which variably approach both the clinician’s knowledge and expertise and the patient’s perspective and knowledge.

In general, disclosing problematic experiences or troubles-telling conveys an affective stance and invites a similar standpoint from the recipient; however, this occurs sometimes with certain situational or institutional restrictions (96–98). Jourard (26) recognised that one’s self-disclosure of problems often leads to another person’s self-disclosure reciprocally. Our data of psychiatric intake interviews did not contain self-disclosures by a clinician in response to a patient’s account. This is generally neither expected nor considered to be appropriate in this institutional context. There are few studies on recipients’ reciprocal responsive self-disclosures to the clients’ self-disclosure in psychotherapy or group counselling (38, 99, 100). These studies report that the speakers’ intimacy and the institutional goal of interaction impact the recipient’s responses to self-disclosure. Revealing negative experiences conveys the speaker’s vulnerability, pushing them to a risky position. Even so, the clients experience relief from physical and emotional tension, advancing additional disclosures to the therapist as well as other familiar people outside of therapy (101). In another context, researchers demonstrated that a storyteller’s emotional load increased the recipient’s physiological arousal level, thus encouraging emotional sharing in affiliative responses, so the teller could be relieved (102). These studies suggest that anyone who shares a story or tells about their own experiences searches for emotional relief and acceptance in mundane or institutional contexts.

Moreover, recent research showed that good patient–provider communication reduces the withholding of sensitive health information in medical encounters (103). Mental service users were afraid of being a “difficult patient”, afraid of stigma or control and discrimination, which impacted their information disclosure, therapeutic relationship and engagement with treatment and services (104–106). However, Tlach and colleagues (107), in their systematic review, showed that the assessment process needs extensive information exchange for treatment decision-making for patients with schizophrenia and depression. These studies support the significant role of clinicians’ orientation and responsiveness to the patients’ disclosure of subjective experiences, which we showed in these particular moments of assessment interviews. Our study is the first to investigate the clinician’s responses to the patient’s self-disclosure of problematic experiences in psychiatric assessment interviews. Let us first consider the clinician’s *agenda question* as a third-position response to the patient’s self-disclosure. On the one hand, it is appropriate and inevitable to ask diagnostically relevant questions during this interview, but on the other, it is not a relevant response to previous self-disclosure at those moments. By posing agenda questions, the clinicians distance themselves from their patients’ negative emotional experiences and similarly distance the patients from their own experiences. Moreover, when the clinician shifts the topic to medical or factual themes, this can imply that the patient’s disclosure is not crucial for their diagnostic work, restricting the patient’s emotional sharing and the possible active elaboration on the patient’s problematic experiences. In this instance, the clinician used their institutional authority against the patient’s urgent and emotionally emphasised account of self-disclosure, causing considerable interactional tension between participants momentarily.

In contrast to the agenda question, we discovered that *follow-up questions* are somehow connected to the patient’s account and knowledge of their experience. However, follow-up questions can approach self-disclosure from two different directions. In the first subgroup, the clinician’s follow-up question selects the topic or asks about the ancillary matter from the patient’s account towards a medical or factual orientation. As a consequence, the patient’s self-disclosure of a problematic experience will not be approached or responded to. By comparison, the follow-up questions in the other subgroup dealt with the patient’s perspective and approached their self-disclosure of a subjective experience, advancing their further elaboration and collaboration between participants.

In *expert interpretations*, the clinician considers the patient’s self-disclosure of a problematic experience. Nonetheless, the clinician transforms its content using his/her expert knowledge. This response type usually serves the diagnostic evaluative work.

In *advice-giving*, the clinician also considers the patient’s self-disclosure. However, the clinician approaches the account indirectly and offers a solution to the patient’s problems. The patient could reject or accept the advice in one way or another, still participating actively. Thus, the advice-giving might advance the patients’ agency or call for an argument for not accepting it.

Finally, *formulation* in the third position legitimates and approaches the gist of the patient’s self-disclosure of a problematic experience from the patient’s personal perspective. The clinician’s formulation displays understanding, explains the patient’s account and relieves emotional tension, which might advance their self-observation and active participation. Using formulation, the clinician becomes more closely acquainted with the patient’s knowledge and emotional condition, which might result in a heightened awareness of their maladaptive action pattern and thus promote the interview process to advance collaboratively.

We presented the distribution of these responsive actions by clinicians to the patient’s self-disclosure in the two types of diagnostic interviews in **Tables 1**, **2**. However, our data allow only indicative comparisons to be made between the two types of interviews. Thus, in the AAU interviews, as responses to the patients’ self-disclosure of problematic experiences, clinicians tend to pose agenda and follow-up questions from a medical/factual orientation and from their expert interpretations. These responses primarily serve the clinician’s diagnostic information gathering based on their expert knowledge and authority. In the DSA interviews, the clinicians tend to mainly use formulations and advice as well as follow-up questions with an experiential or medical/factual orientation. These responses approached the patient’s knowledge and emotional states of adverse experience from their personal perspective directly or indirectly, paving the way to advance the patient’s self-observation and to build their working relationship collaboratively.

The different third-position responsive actions after the patients’ self-disclosure characterised the speakers’ relation to each other’s knowledge. In our results, some response types revealed the clinician’s orientation to medical/factual diagnostic evaluation, distancing themselves from lifeworld experiences. Other clinician’s responses are oriented closely to the patient’s disclosed experiences by dealing with and elaborating on them. Regarding speakers’ orientations, Levinson (108, p. 127) has observed that “*actions often form a part of a larger project inheriting part of their import from the larger whole*”. While the clinicians in the DSA interviews were actively oriented predominantly towards the patient’s lifeworld adverse experience, in the AAU interviews, they were primarily oriented towards gathering diagnostic information. Levinson claimed that speakers are sometimes unaware of each other’s larger projects. Previously, Savander and colleagues (56) found that in their larger project, while the patients were usually oriented to sharing and complaining about adverse lifeworld experiences, the clinicians were primarily oriented to gathering diagnostic information. These divergent interactional projects occasionally clashed, causing considerable interactional tension between participants momentarily.

Although clinicians’ reciprocal self-disclosure is inappropriate in this type of institutional encounter, the DSA approach facilitates responsiveness to the patient’s account. In the DSA interviews, the clinicians’ responses oriented to and addressed the patient’s self-disclosure of a problematic experience, thus advancing the self-observation and building actively and collaboratively the clinician–patient working relationship. On the other hand, in the AAU interviews, the clinicians’ response types might occasionally have created considerable interactional tension, at least when the clinicians posed an agenda question and moved through it towards another topic, distancing themselves from the patient’s self-disclosure of adverse experiences. While agenda questions are inevitable interview techniques in any psychiatric diagnostic interview, the clinician must be aware of when and where it is appropriate to use them without ignoring the patient’s self-initiative disclosure of problematic experiences. Indeed, any information gathering defeats its purpose when the participants’ therapeutic or working relationships lack a sufficient amount of purposeful collaboration.

During the last decades, new ways to advance the patient’s involvement and partnership in their personal recovery process have been sought in mental healthcare. Increasingly, the patient is considered as an expert of their own experiences and an equal partner with professionals (109–114). In psychotherapy research, the therapeutic or working alliance is studied as a purposeful collaboration with an affective bond between participants (59, 60, 115). The therapeutic or working alliance is an operationalised and measurable phenomenon and thus might advance quantitative studies on the value-based personal recovery process between the clinician and the patient (116). In our original comparative study (54), we found that the working alliance congruence between participants was higher in the DSA interviews, as compared to the AAU approach.

Moreover, previous studies have reported that the third-position responses have a decisive and characterising institutional role in maintaining and moving the interaction forward in educational or medical and psychotherapeutic environments (49–51). These significant moments in psychiatric interviews revealed that the third-position responses convey the clinician’s orientation and represent a decisive function in regulating diagnostic information gathering and in establishing a therapeutic relationship. The patient’s self-disclosure invites acceptance and understanding from the clinician, may offer important information for diagnostical assessment and may promote the co-construction of an individual psychological case formulation as possible obstacles to their agency and recovery.

### 4.1 Limitations

Our study has some limitations. Firstly, the data were selected from previous studies of only 10 matched psychiatric interviews (61, 62). This means that regardless of the careful procedures and interrater process, this relatively small sample size may have biassed the results. In addition, we simplified the data analysis for quantification by adopting Mishler’s binarity when selecting the speakers’ orientations towards medical/factual or experiential realms. In addition, these analysed trajectories do not demonstrate all the details and information exchange of the complete interviews, even though they provide relevant information regarding the interactional projects and orientations of the clinicians and patients. Although all clinicians had several years of work experience, their differing expertise and training in the AAU or DSA group might have influenced the results somewhat.

In the future, further studies need to focus on more detailed examinations of entire recorded interviews. In addition, it would be relevant to adopt CA to investigate the clinicians’ practices*—*questions and responses*—*when they use the DSA approach and purposefully invite and approach the patient’s adverse experiences in the clinicians’ attempt to complement their diagnostic work with an individual psychological case formulation. Examining the dynamic development of DSA case formulation during the assessment period using CA would be important in the future. We suggest that more interactional and quantitative studies should be carried out into the effectiveness of the DSA approach with larger groups of patients with different mental disorders.

### 4.2 Conclusion

When patients disclose their subjective negative experience, they highlight the intensity and urgency of their experiences. This study suggests that the clinician’s third-position response type basically defines what the clinician as an expert takes up from the patient’s telling. In other words, whether clinicians distance themselves from their patients’ subjective experience or approach their experience, they regulate participants’ orientation towards both the medical/factual and experiential realm, adjusting the patient’s involvement and active participation in the assessment and treatment. In naturally occurring and unstructured psychiatric interviews, the clinician can observe and recognise the patient’s self-disclosure of their lifeworld experience. By approaching the patient’s self-disclosure, the clinician might relieve the patient’s suffering and advance their self-observation and active participation, all of which strengthen their agency regarding the treatment of their mental disorder and their recovery. These interactional moments are noticeable and noteworthy and help co-construct a psychological case formulation step by step and side by side with the diagnostic categories.

## Data availability statement

The datasets presented in this article are not readily available because the Ethics Committee of Tampere University Hospital does not allow the transfer of audio-recorded data to third parties. Requests to access the datasets should be directed to the corresponding author.

## Ethics statement

The studies involving humans were approved by the Ethics Committee of Tampere University Hospital (R 14094). The studies were conducted in accordance with the local legislation and institutional requirements. The participants provided their written informed consent to participate in this study.

## Author contributions

ES: Writing – original draft, Visualization, Validation, Resources, Project administration, Methodology, Investigation, Funding acquisition, Formal analysis, Data curation, Conceptualization. LV: Writing – review & editing, Validation, Supervision, Methodology, Investigation. JH: Writing – review & editing, Validation, Supervision, Resources, Investigation, Data curation. AP: Writing – review & editing, Validation, Supervision, Methodology, Investigation, Formal Analysis, Data curation, Conceptualization.

## Appendix

Transcription symbols—adapted from Jefferson (58)

AB:  Speaker identification: doctor (DO), patient (PA), psychologist (PS)

→  Line containing phenomenon discussed in text

[]  Overlapping talk or anonymisation

=  No space between turns

(.)  A pause of less than 0.2 s

(1.0)  Pause: silence measured in seconds and tenths of a second

°word°   Talk lower volume than the surrounding talk

WORD  Talk louder volume than the surrounding talk

.hh  An in-breath

hh  An out-breath

mt, tch, krhm  Vocal noises

£word£  Spoken in a smiley voice

@word@  Spoken in an animated voice

#word#  Spoken in a creaky voice

wo(h)rd  Laugh particle inserted within a word

((word))  Transcriber’s comments

()  Transcriber could not hear what was said

word  Accented sound or syllable

-  Abrupt cutoff of preceding sound

:  Lengthening of a sound

>word<  Talk faster than the surrounding talk

<word>  Talk slower than the surrounding talk

word<  Sharp tone at the end of a word

↑↓  Rise or fall in pitch

?  Final rise intonation

,  Final level intonation

.  Final falling intonation
